# An Aptitude for Altitude: Are Epigenomic Processes Involved?

**DOI:** 10.3389/fphys.2019.01397

**Published:** 2019-11-22

**Authors:** Colleen G. Julian

**Affiliations:** Division of Biomedical Informatics and Personalized Medicine, Department of Medicine, University of Colorado School of Medicine, Aurora, CO, United States

**Keywords:** evolution, integrative, physiology, altitude, epigenome

## Abstract

In recent years, high-throughput genomic technologies and computational advancements have invigorated efforts to identify the molecular mechanisms regulating human adaptation to high altitude. Although exceptional progress regarding the identification of genomic regions showing evidence of recent positive selection has been made, many of the key “hypoxia tolerant” phenotypes of highland populations have not yet been linked to putative adaptive genetic variants. As a result, fundamental questions regarding the biological processes by which such adaptations are acquired remain unanswered. This Mini Review discusses the hypothesis that the epigenome works in coordination with underlying genomic sequence to govern adaptation to the chronic hypoxia of high altitude by influencing adaptive capacity and phenotypic variation under conditions of environmental hypoxia. Efforts to unravel the complex interactions between the genome, epigenome, and environmental exposures are essential to more fully appreciate the mechanisms underlying human adaptation to hypoxia.

## Introduction

“Each living organism has two histories that determine its biology: an evolutionary history whose duration is in the hundreds of thousands of years, and a developmental history that starts at the time of conception.”

Ze’ev Hochberg

Oxygen homeostasis, a process on which human survival depends, is severely challenged by the hypobaric hypoxia of high altitude. Despite this challenge, humans have lived and thrived at high altitudes on the Qinghai-Tibetan Plateau (western China), the Andean Altiplano (South America) and the Semien Plateau (Ethiopia) for millennia (Aldenderfer, 2011; Scheinfeldt et al., 2012; Huerta-Sanchez et al., 2013; Jeong et al., 2014; Rademaker et al., 2014). These highland populations possess an array of “hypoxia-tolerant” physiological traits and exhibit strong genomic signals of recent positive selection within or near genes involved in oxygen sensing or hypoxic response (Beall et al., 2010; Bigham et al., 2010, 2014; Simonson et al., 2010; Yi et al., 2010; Scheinfeldt et al., 2012; Huerta-Sanchez et al., 2013; Zhou et al., 2013; Cole et al., 2014; Crawford et al., 2017). Collectively, these adaptive signals point toward genetic adaptation as a predominant molecular mechanism by which humans have adapted to the high altitude environment. Yet, the adaptive variants identified do not explain the full range of hypoxia-tolerant features characteristic to highland populations and their relevance for improving reproductive success or sexual selection remains unclear. In this context, it is vital to emphasize that natural selection acts not on genotypes directly, but rather phenotypes which most often are the product of gene-gene interaction, gene-environment interaction and non-sequence-based features of the genome (e.g., epigenetic marks) that are critical for transcriptional regulation in response to environmental and developmental triggers. This complexity underscores the need to emphasize more dynamic models of human adaptation than have traditionally been appreciated (Box 1).

**BOX 1. Models of human adaptation.**

Historically, models of human adaptation have partitioned the process of adaptation into distinct components that differ in timescale, stability, and method of acquisition. On the most transitory end of the continuum are comparatively rapid molecular and physiologic changes that maintain homeostasis under shifting environmental or biological exposures. Conversely, stimuli experienced during critical developmental periods may cause physiologic or morphologic changes that persist into later life, a phenomenon termed developmental adaptation or plasticity. On the most stable side of the “adaptive spectrum” is natural selection. Natural selection refers to the increased frequency of genetic variants within a population as a result of their contribution to phenotypes that improve reproductive success or sexual selection. Recent work suggests that the process of human adaptation requires a more dynamic interaction of the various modes of adaptation than previously appreciated. Epigenetic processes are vital for mediating transcriptional and physiological responses to hypoxia. Physiologic responses, however, can influence epigenetic patterns. Epigenetic events also play a critical role in determining cellular identity and are involved in the effect of early life exposures to alter developmental trajectories. While epigenomic marks exert a powerful influence on the way genomic sequences are translated into phenotypic traits, genetic background can also influence the probability of epigenetic modification. Lastly, epigenetic marks can facilitate changes to genetic sequence via several mechanisms, including chromosomal recombination and cytosine to tyrosine transitions resulting from the deamination of methylated CpG motifs. From this vantage point, elements of the adaptive process are highly.

While genomic sequences are effectively hardwired, dynamic epigenetic changes to chromatin conformation determine regions of transcriptional silence or transcriptional potential in response to biological or environmental cues to produce diverse cellular phenotypes and functions (Bird, 2007; Feinberg, 2007; Bonasio et al., 2010). Several unique epigenetic mechanisms exist, including cytosine methylation, histone modification and variants, and RNA-based mechanisms. This Mini Review focuses on cytosine methylation, defined by the addition of a methyl group to the C-5 position of cytosine residues within CpG dinucleotides, given its prominent role in genomic imprinting, transcriptional regulation, and the silencing of repetitive DNA elements and comparatively extensive study in humans (Robertson, 2001; Jones and Baylin, 2002). A comprehensive review of epigenetic mechanisms underlying transcriptional responses to hypoxia is provided elsewhere (Choudhry and Harris, 2018).

Across the human genome, CpG motifs are extensively methylated (Saxonov et al., 2006). Dispersed among these, however, are regions rich with CpG dinucleotides occurring in sequence (“CpG islands”) that typically remain unmethylated. Approximately 70% of annotated gene promoters in the human genome are associated with a CpG island (Saxonov et al., 2006). CpG island hypomethylation typically promotes transcription factor binding and active gene transcription, whereas hypermethylation often inhibits transcription factor binding and thereby establishes an inactive chromatin state (Feinberg, 2007). Exceptions do exist, however, making it difficult to simplify the complex interrelationship of these epigenetic events. In this way, the epigenome exerts a powerful influence on the translation of genomic sequences to phenotypic traits (Feinberg, 2007; Bonasio et al., 2010). For instance, the differentiation of pluripotent cells, each with an identical DNA sequence, into phenotypically distinct cell types is driven by epigenetic processes (Reik, 2007). In other words, non-sequence based changes to chromatin conformation enable phenotypic flexibility about a given genotype.

This Mini Review considers that epigenomic mechanisms may contribute to human adaptation to the high altitude environment in a least four interconnected ways including (1) the modification of transcriptional or phenotypic flexibility under conditions of limited oxygen availability, (2) enhancing (or limiting) phenotypic variation and thereby influencing the heritability of traits, (3) underlying genetic variation affecting local or distant methylation state, and (4) epigenetic silencing to mask deleterious dominant mutations or reveal recessive mutations. While still speculative and theoretical, the concept that epigenomic processes contribute to heritable phenotypic variation is gathering momentum.

## Epigenetic Regulation of Hypoxia Responsive Transcriptional Programs

The hypoxia-inducible transcription factor (HIF), consisting of two a-subunits (HIF1/2a) and a constitutive b-subunit, is the predominant regulator governing robust transcriptional responses to hypoxia that collectively act to defend oxygen homeostasis (Semenza, 2011). Under conditions of adequate oxygen supply, the hydroxylation of the HIF1/2a subunits via prolyl hydroxylases (PHDs) (Bruick and McKnight, 2001) facilitates the binding of von Hippel–Lindau tumor suppressor (vHL) and, in turn, the proteasomal degradation of HIF1/2a (Ivan et al., 2001). In contrast, under hypoxic conditions, the hydroxylation of HIF1/2a by PHD is impaired, allowing HIF1/2a to escape recognition by vHL and translocate to the nucleus. Subsequently, HIF1/2a dimerizes with HIF1-b and subsequently binds with hypoxia-responsive elements (5′ A/GCGTG 3′) and associated cofactors across the genome to initiate the HIF-transcriptional program and cellular adaptation to hypoxia (Majmundar et al., 2010).

Epigenetic silencing of genes integral for the stabilization of HIF, such as vHL and EPAS1, has been shown to be vital for mediating the transcription of HIF pathway genes *ex vivo* in isolated primary cells or cell lines (Herman et al., 1994; Lachance et al., 2014). DNA methyltransferase 3a (DNMT3A), for example, prompts the *de novo* CpG methylation of the EPAS1 promoter, an effect that inhibits HIF2α-regulated gene transcription (Lachance et al., 2014). Conversely, defective DNMT3A inhibits the epigenetic silencing of EPAS1, resulting in unscheduled EPAS1 activation (Lachance et al., 2014). Further underlining the likely critical role of epigenetic processes at high altitude, the induction of erythropoietin (EPO) gene expression is governed by methylation events within the promoter and 5′-untranslated region of the EPO gene. EPO is considered to be the “master regulator” of red blood cell production. Hypoxia also alters methylation status by regulating the expression of enzymes that catalyze methylation or demethylation events [e.g., DNMTs and ten-eleven-translocation (TET) 2, respectively] (Thienpont et al., 2016). TET2 enzymes induce DNA demethylation by hydroxylating 5-methylcytosine (5 mC) to 5-hydroxymethylcytosine (5 hmC) which, after a series of oxidation events, is ultimately substituted with an unmodified cytosine by base-excision repair to achieve demethylation (Tahiliani et al., 2009; Ito et al., 2011). Given that TET2 and 5 hmC are known to be modified by hypoxia in the context of human disease, (Choudhry and Harris, 2018) demethylation events also likely contribute to the epigenetic regulation of transcriptional responses to the chronic hypoxia of high altitude.

Histone acetyltransferases and demethylases also contribute to the regulation of chromatin conformation within and around HIF-binding sites and are regulated, in part, by hypoxia (Kallio et al., 1998; Wellmann et al., 2008; Watson et al., 2009). Histone deacetylases, for instance, are reported to increase HIF-1α protein stability, thereby promoting HIF-1 transactivation or, in the case of histone deacetylase 7, to augment HIF-1 transcriptional activity via physical interaction with HIF-1α, p300, and CBP (Kato et al., 2004). Further studies are required to determine whether the regulatory role of epigenetic processes for transcriptional responses to hypoxia observed *ex vivo* occur similarly *in vivo*.

## Differential Methylation With High-Altitude Exposure

Recent reports document differential methylation patterns in highland populations in association with the duration of high-altitude residence and phenotypes presumed to be maladaptive at high altitude. One study contrasting EPAS1 promoter and LINE-1 repetitive element methylation among Andean Quechua residing at high altitudes (4300 m) or sea level in Peru suggests that altitude of current residence and lifetime exposure to high altitude is inversely related to EPAS1 methylation and directly associated with LINE-1 methylation (Childebayeva et al., 2019). The study does not report whether association between methylation status and adaptive phenotypes existed (Childebayeva et al., 2019). In the peripheral blood mononuclear cells of Andean men living in La Paz-El Alto, Bolivia (3600 to 4000 m), a recent report identified several differentially methylated regions (DMR) at base-pair resolution in individuals with excessive erythrocytosis (EE), considered to be a maladaptive response to chronic hypoxia, compared to healthy, age and altitude-matched controls (Julian, 2017). The most notable DMR identified was a hypermethylated region within EGLN1, (Julian, 2017) the gene that encodes PHD2 – an enzyme that is vital for inhibition of HIF-regulated gene transcription. Notably, the DMR identified was located within the CpG island that surrounds the EGLN1 promoter and was in close proximity (700 bp) to an EGLN1 SNP (rs12097901) that occurs at an increased frequency in Tibetans (Lorenzo et al., 2014; Tashi et al., 2017) In Tibetan populations, the rs12097901 SNP is inversely related to hemoglobin concentration and has been reported to affect PHD2 binding, yet the latter remains a topic of discussion (Bigham and Lee, 2014) rs12097901 also exists in Andean populations, although at a reduced frequency, indicating that the adaptive benefits afforded by this particular SNP may also be of potential importance for individuals of other high-altitude populations. If specific epigenomic marks or the genetic potential for epigenetic regulation are heritable and confer a selective advantage (or disadvantage) under conditions of limited oxygen availability it is tempting to speculate that such effects may contribute to human adaptation to high altitude.

## Evidence for Epigenomic Heritability

**FIGURE 1:**
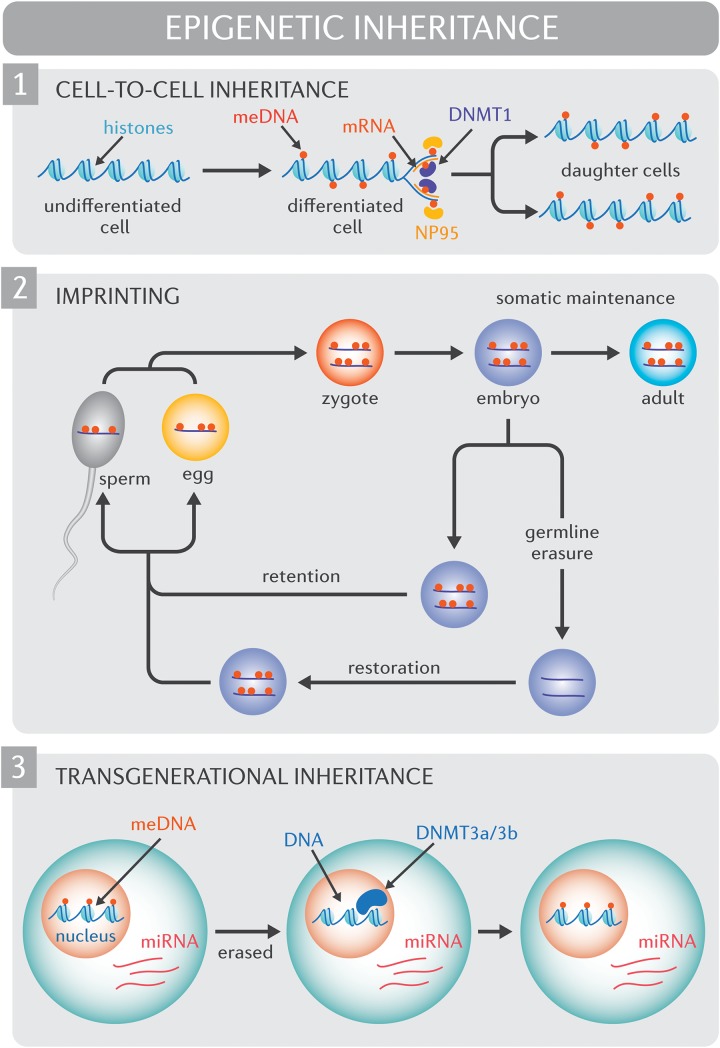
Molecular mechanisms of epigenetic inheritance. (1) Cell-to-cell inheritance of epigenetic modifications. At the outset of semi-conservative DNA replication, cytosine methylation marks (red circles) are only present on the original (“mother”) strands of DNA. NP95 (yellow ovals) binds methylated cytosines, and recruits DNMT1 (purple oval) which is responsible for the *de novo* methylation of DNA on the complementary daughter DNA strands. (2) Genomic imprinting. Epigenetic mechanisms drive genomic imprinting, a process by which select genes are expressed according to parental heritage. In the pre-implantation embryo, the widespread erasure of epigenomic marks spares imprinted genes (red circles, DNA methylation of imprinted genes) such that the epigenome of imprinted genes persists into adulthood. Extensive epigenetic reprograming of non-imprinted genes and select imprinted genes also occurs in the germ line. Epigenetic modifications that are not retained through the reprograming process are subsequently restored in a sex-specific manner. (3) Transgenerational inheritance. Non-imprinted genes undergo vast, albeit incomplete, epigenetic erasure prior to implantation. During this process, much of the epigenome, including DNA methylation marks (meDNA) are “erased”. It has been proposed that small and long non-coding RNA species (including micro RNAs [miRNA]) recruit methylating enzyme complexes such as DNMT3a/3b to restore epigenome to its original form.

To be pertinent for human adaptation, epigenomic diversity or the capacity for epigenetic diversity must be propagated. Understanding how environmental exposures impact the human epigenome, and whether or how such effects may be inherited continues to be an area of intense investigation (Kaati et al., 2002; Heijmans et al., 2008; Bygren et al., 2014). At this point, it is essential to distinguish between cell-to-cell inheritance, intergenerational inheritance, and transgenerational inheritance. Cell-to-cell epigenetic inheritance, or mother-daughter cell transmission, is well established (Figure 1) and reviewed in detail elsewhere (Probst et al., 2009). Intergenerational inheritance refers to the transmission of epigenetic traits across a single generations (F0 to F1), while transgenerational inheritance spans two or more generations (F0 to F2 onward). Inter- and transgenerational epigenetic heritability in mammals continues to be a contentious debate, predominantly due to the fact that non-imprinted genes undergo vast, albeit *incomplete*, epigenetic erasure before implantation (Smallwood et al., 2011; Gkountela et al., 2015; Guo et al., 2015; Tang et al., 2015). It follows that for intergenerational inheritance to occur, epigenetic marks in the preimplantation embryo would need to evade extensive reprograming. It is worth noting, however, that arguments against intergenerational and transgenerational epigenetic heritability rely heavily on the assumption that epigenetic marks are inherited in a replicative rather than a reconstructive manner. Mechanisms do exist to protect against the reprograming of specific sequences (Skvortsova et al., 2018). For instance, epigenetic processes drive genomic imprinting, or the expression of specific genes in a parent-of-origin manner. Specifically, DNA methylation events epigenetically silence the inactive allele and these changes are resistant to post-fertilization epigenetic methylation reprograming.

For true transgenerational epigenetic inheritance to occur, epigenetic marks would need to avoid erasure in primordial germ cells, a phenomenon that has been observed in mammals (Probst et al., 2009). While evidence supports the persistence of epigenetic states across generations (Roemer et al., 1997; Anway et al., 2005; Rexhaj et al., 2011) and transmission between somatic cells and germ cells, (Hitchins and Ward, 2009) the regulatory processes underlying these observations remain unclear. Small RNAs and long non-coding RNAs are one potential mechanism for the “inheritance” of epigenomic marks beyond a single cell lineage (Cech and Steitz, 2014; Holoch and Moazed, 2015). In particular, recent reports reveal that somatic epigenetic modifications may be transferred to the gamete by regulatory RNA species that direct histone modification or methylation status in a sequence-specific manner rather than being transferred intact (Figure 1; Chen et al., 2016; Zhang et al., 2018). Through this same mechanism, epigenetic marks could either bypass the widespread epigenetic reprograming that occurs in early development or subsequently “reconstruct” epigenetic patterns after the reprograming process. Constitutional epialleles, defined as epigenetic marks deriving from the early embryo or parental germline, constitute another mechanism for transgenerational epigenetic inheritance, however the stability of constitutional epialleles across meiotic division remains unclear (Hitchins et al., 2007).

While epigenetic patterns vary extensively between populations, the extent to which underlying genetic architecture drives these differences remains unclear. Current literature indicates that a comparatively large proportion of population-specific CpG methylation patterns can be attributed to underlying genetic variation (Fraser et al., 2012; Heyn et al., 2013; Moen et al., 2013). Environmental influences, however, also appear to exert a powerful influence given that, in these comparative epigenomic studies, no direct relationship to genetic variation could be detected for approximately one-third to one-half of the differentially methylated loci (Fraser et al., 2012; Heyn et al., 2013; Moen et al., 2013). Heritable variations with respect to CpG density may permit or prohibit methylation marks that govern transcriptional responses to hypoxia. While only a small proportion of cytosine methylation marks across the genome can be attributed to *cis*- and *trans*-acting single nucleotide polymorphisms (SNPs), SNPs that disrupt (or create) CpG motifs heavily influence methylation status of local and distant (<10 kb) CpG sites and, in turn, the capacity for epigenetic regulation of gene expression (Zemach et al., 2010; Bell et al., 2011; Taudt et al., 2016). In other words, epigenetic regulation of gene transcription would theoretically be constrained by reduced CG content and more permissive by increased CG content. Illustrating that such processes could potentially contribute to high-altitude adaptation, a recent report found that nearly 40% of *EPAS1* SNPs showing evidence of positive selection in highland populations altered CpG content (Julian, 2017). Given that the *EPAS1* promoter is encompassed by a CpG island and is transcriptionally regulated via epigenetic mechanisms, (Lachance et al., 2014) these findings are of particular interest.

## Potential Evolutionary Consequences of Epigenomic Variation

Epigenomic variation may affect evolutionary processes in several different ways. For one, epigenetic mechanisms modify phenotype, the subject of natural selection, and can thereby influence the heritability of traits. Second, phenotypic variation afforded by epiallelic silencing or other genetic mechanisms may afford greater flexibility under rapidly changing environmental or biological conditions, (Feinberg and Irizarry, 2010) such as pregnancy and exercise, that pose significant challenges for oxygen homeostasis, particularly at high altitude. Third, using mathematical models, it has been proposed that heritable genetic variants, in particular, the loss or gain of CpG motifs, that enhance phenotypic diversity likely improve reproductive fitness, and that such effects may be significant for evolutionary adaptation to shifting environmental conditions (Feinberg and Irizarry, 2010). In support of this concept, short-term, fluctuating evolution appears to occur without cumulative change. Specifically, the analysis of evolutionary rates measured over 10^0^–10^7^ generations revealed a marked pattern of constrained phenotypic fluctuation, such that the predicted phenotypic variation of samples separated by ten generations is effectively equivalent to that of samples separated by one million generations (Gingerich, 2001; Estes and Arnold, 2007). Fourth, epigenetic marks can promote changes to genetic sequence by several mechanisms, including cytosine to tyrosine transitions due to the deamination of methylated CpG motifs, and chromosomal recombination (Janion, 1982; Sved and Bird, 1990; Carbone et al., 2009). Methylated CpG motifs, for instance, convert to TpG at a rate 10 to 50 times greater compared to different transitional changes (Branciamore et al., 2014). Methylation also reduces the possibility of recombination and recombination-based repair (Xia et al., 2012). Finally, epigenetic silencing could also conceal dominant deleterious mutations or increase the probability for fixation of recessive mutations (Chess, 2012). These observations, together with accumulating evidence supporting the heritability of epigenetic marks, raise new questions about how the genome orchestrates the unique physiologic attributes of highland populations.

## Summary and Future Prospects

Promising investigations are poised to begin unraveling the molecular mechanisms by which genomic signals showing evidence of natural selection confer purportedly advantageous phenotypes of highland populations within the context of the high-altitude environment. At present, however, the adaptive variants identified by single nucleotide polymorphism (SNP) array or case-control comparisons do not explain the full range of hypoxia-tolerant features characteristic to highland populations, emphasizing the need for whole-genome sequencing and a fuller understanding of the degree to which non-sequence based features of the genome contribute to the adaptive physiological features observed.

Epigenomic studies remain challenging. Human epigenomic studies, for instance, are limited, in part, by the cell-specific nature of epigenetic marks. Unlike genetic variation, epigenetic patterns vary across cell types and, in humans, it is often not possible to acquire the tissue of interest, let alone pure cell populations from that tissue. Therefore, surrogate cells or tissues must be carefully considered, and investigators should be certain to acknowledge that, in such cases, study results may not reflect the epigenetic status of the target organ. Furthermore, underlying DNA sequence variations, which are well-established and potent contributors to the inheritance of epigenetic states, are often disregarded in epigenome-wide association studies, (Lappalainen and Greally, 2017) emphasizing the need for whole-genome sequencing and an integrated genomic-epigenomic approach. Finally, while functional investigations of site-specific methylation state remain difficult, the introduction of genome-editing techniques including the CRISPR/Cas-9 system and Transcription Activator-Like Effector Nucleases (TALENs) permits focused CpG methylation and demethylation *in vitro* and *in vivo* (Maeder et al., 2013; Bernstein et al., 2015). Using these techniques, experimental animal studies could evaluate whether altering the methylation state of targeted CpG sites within specific cell types affects transcriptional and physiological responses to hypoxia *in vivo*. In recent years, sequencing technologies and analytical capabilities have vastly expanded knowledge regarding the mechanisms governing human variation and disease and provide the opportunity to gain a deeper understanding of the genome-epigenome interaction and its relevance for human adaptation and adaptive potential.

## Author Contributions

CJ wrote the manuscript and approved the submitted version.

## Conflict of Interest

The authors declare that the research was conducted in the absence of any commercial or financial relationships that could be construed as a potential conflict of interest.
